# Sound stream segregation: a neuromorphic approach to solve the “cocktail party problem” in real-time

**DOI:** 10.3389/fnins.2015.00309

**Published:** 2015-09-02

**Authors:** Chetan Singh Thakur, Runchun M. Wang, Saeed Afshar, Tara J. Hamilton, Jonathan C. Tapson, Shihab A. Shamma, André van Schaik

**Affiliations:** ^1^Biomedical Engineering and Neuroscience, The MARCS Institute, University of Western SydneySydney, NSW, Australia; ^2^Department of Electrical and Computer Engineering and Institute for Systems Research, University of MarylandCollege Park, MD, USA

**Keywords:** temporal coherence, cochlea, cocktail party problem, FPGA, machine-based speech recognition

## Abstract

The human auditory system has the ability to segregate complex auditory scenes into a foreground component and a background, allowing us to listen to specific speech sounds from a mixture of sounds. Selective attention plays a crucial role in this process, colloquially known as the “cocktail party effect.” It has not been possible to build a machine that can emulate this human ability in real-time. Here, we have developed a framework for the implementation of a neuromorphic sound segregation algorithm in a Field Programmable Gate Array (FPGA). This algorithm is based on the principles of temporal coherence and uses an attention signal to separate a target sound stream from background noise. Temporal coherence implies that auditory features belonging to the same sound source are coherently modulated and evoke highly correlated neural response patterns. The basis for this form of sound segregation is that responses from pairs of channels that are strongly positively correlated belong to the same stream, while channels that are uncorrelated or anti-correlated belong to different streams. In our framework, we have used a neuromorphic cochlea as a frontend sound analyser to extract spatial information of the sound input, which then passes through band pass filters that extract the sound envelope at various modulation rates. Further stages include feature extraction and mask generation, which is finally used to reconstruct the targeted sound. Using sample tonal and speech mixtures, we show that our FPGA architecture is able to segregate sound sources in real-time. The accuracy of segregation is indicated by the high signal-to-noise ratio (SNR) of the segregated stream (90, 77, and 55 dB for simple tone, complex tone, and speech, respectively) as compared to the SNR of the mixture waveform (0 dB). This system may be easily extended for the segregation of complex speech signals, and may thus find various applications in electronic devices such as for sound segregation and speech recognition.

## Introduction

Humans can segregate sound sources and focus their attention on specific sounds, while filtering out a range of other background sounds with ease (Bregman, 1990). This attentional ability is known as the “cocktail party effect” (Cherry, 1953), for it enables one to focus on a single conversation in a noisy room. Intentions and attention play a key role in segregating complex auditory scenes into foregrounds and backgrounds, by directing sensory and cognitive processes to pertinent auditory features (Woldorff et al., 1993; Shinn-Cunningham, 2008; Elhilali et al., 2009b). Various acoustic characteristics of sound such as pitch, frequency, timbre, and spatial location may be the focal point of auditory attention in selective hearing (Lee et al., 2012).

The human auditory system is a highly efficient and sensitive sensory system. Sound waves collected in the outer ear travel through the middle ear to reach the cochlea, which serves as the front-end of the auditory system (Guinan et al., 2012). Different locations on the basilar membrane (BM) of the cochlea vibrate in response to specific sound frequencies, thus enabling the cochlea to function as a frequency spectrum analyser (Gold and Pumphrey, 1948; Plomp, 1964). The mechanical vibrations are transduced by the inner hair cells into neural impulses along the auditory nerve (LeMasurier and Gillespie, 2005). Subsequent processing in the brain includes pitch perception for complex tones (Hall and Plack, 2009), sound localisation (Grothe et al., 2010), sound segregation (Carlyon, 2004) and identification (Alain et al., 2001).

Machine-based speech recognition systems have so far not been able to match the functional efficiency of biological auditory systems (Lyon, 2010). It is especially desirable to develop a machine-based auditory system with the ability to segregate sound sources. Such systems would have a large number of applications such as speech recognition in a noisy background, source localisation, sound-based human computer interaction, the design of autonomous robots with the ability to hear and respond to sounds, mobile devices that can seamlessly use voice commands and the design of intelligent hearing aids, as sound segregation rapidly deteriorates in hearing impaired individuals. Several computational models have been proposed to solve the cocktail party problem of speech recognition in a noisy environment (Cooke and Ellis, 2001; Cooke et al., 2010; Shao et al., 2010; Shamma et al., 2011). However, all of these are software models and highly computationally intensive, which cannot process sound in real-time.

Here, we utilize a temporal coherence model of sound stream segregation (Krishnan et al., 2014) and adapt it for hardware implementation in a Field Programmable Gate Array (FPGA). The model works on the principle of temporal coherence (Elhilali et al., 2009a), meaning that the different types of features (e.g., pitch, location, loudness, etc.) belonging to a sound source fluctuate in strength at exactly the same times, while those belonging to different sound sources are rarely synchronized. The model also incorporates the feature that neural response patterns generated by the auditory features of a sound source are highly correlated (Shamma et al., 2011). Together, these principles allow separation of target speech from background noise using attentional mechanisms. A unique feature of the temporal coherence model is that it does not require any training or prior knowledge of target signal and background noise. Further, it is worth mentioning that since this model is highly computationally intensive, an FPGA implementation that runs in real-time is useful.

The temporal coherence model consists of two stages—feature extraction and clustering (Krishnan et al., 2014). The feature extraction stage employs an electronic cochlea along with rate filters. For the hardware implementation, we employ a neuromorphic model of the cochlea called CAR-FAC (Cascade of Asymmetric Resonators with Fast-Acting Compression) (Lyon, 2011). We have previously implemented the BM module of the CAR-FAC model in an FPGA (Thakur et al., 2014a). Here, we have further improved the FPGA implementation by incorporating a simplified inner hair cell module in addition to the BM module. This electronic cochlea, with the BM, and inner hair cell modules, extracts the auditory features of input sound stimuli. The rate filters then carry out a multi-resolution analysis of the cochlear output. The output of each rate filter is referred to as a channel. In the clustering stage, correlation among the channels is computed to identify coherent features, and the attention signal is utilized to select target features that serve as a mask for segregating and reconstructing source of interest.

We have tested our sound segregation model by using an alternating-tone sequence and an alternating-harmonic sequence in the hardware model, and a mixture of alternating speech in the software model. The FPGA system was able to segregate the target sound stream from the mixture of sounds in real-time. Our work demonstrates that the temporal coherence model of auditory filtering can be implemented on an FPGA for segregation of sound sources in real-time. The FPGA implementation of the temporal coherence model described here may find applications in various machine-hearing applications. This paper is organized as follows: the computational model and the system architecture are described in the Materials and Methods Section. Sound segregation from pure tone mixtures, complex tone mixtures and speech mixtures are presented in the Results Section, which is followed by the Discussion Section.

## Materials and methods

### Temporal coherence model of auditory streaming

We have used a biological plausible temporal coherence model for our hardware implementation (Krishnan et al., 2014). This model exploits two characteristics of a sound source for auditory filtering—first, the acoustic features of a sound source are coherently modulated in a temporal manner, and second, the neural patterns generated in response to a sound source are highly correlated. The guiding principle, based on one of the Gestalt Principles, is that auditory channels highly correlated over a short time period represent a common fate (Bregman, 1990; Blake and Lee, 2005). This algorithm does not require any training or prior knowledge of the sound sources. This model also employs attention to specific attributes of a source to segregate it from the background. The model comprises of two stages:

#### Feature extraction stage

First, a cochlear model is used to compute an auditory spectrogram of the input sound. Next, features extracted from the cochlear stage are gone through a temporal analysis with multi- rate filters. The filters are selective to different temporal modulation rates ranging from slow to fast (2, 4, 8, 16 Hz), covering the cortical time-scale. In the current FPGA solution, we have implemented only one rate filter of 4 Hz, since it is sufficient for the tested sound mixtures. We can easily extend our system to multi-rate filters, and as the rate filters work in parallel, increasing their number will not affect the performance of the system.

#### Clustering stage

In the actual model, a correlation matrix is calculated by computing the outer product of the multidimensional channels (output of the rate filters), and is updated for each time-step. The pair-wise correlation between channels is indicative of their degree of synchrony. The next process is to employ an attention signal to select correlation coefficients for the target stream, which then act as a mask to segregate and reconstruct the target stream from uncorrelated streams. In our system, we use an attention signal as an input, which avoids the calculation of the correlation matrix and reduces the computational burden (Section Attention Signal and Mask for Reconstruction). The attention signal is an exclusive feature present only in the target stream and it is used computationally to facilitate the identification of the target stream. The attention signal acts as an anchor to segregate the target stream and this anchor could be a pitch signal, an envelope of the target speech, or an envelope of lip movement etc.

### Design methodology

**Figure 1:**
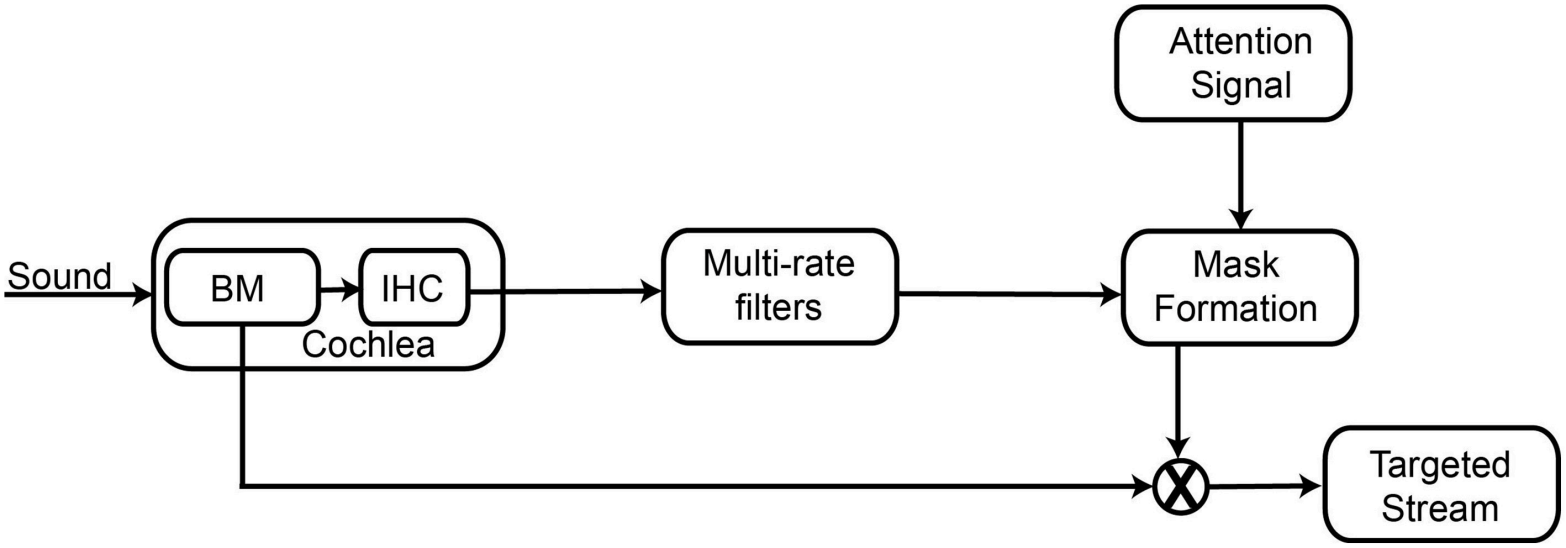
**Schematic of the FPGA implementation of the temporal coherence model of sound segregation**. The cochlea receives the auditory input, and transforms the sound signal into a frequency spectrum. The cochlear output of each frequency channel is passed on to a rate filter that performs a temporal analysis of the rate of variation of the amplitude of the cochlear output at that frequency channel. An externally determined attention signal, which is temporally correlated with the target stream, is correlated with the output of the rate filters to create a mask for stream segregation. This mask is used with the output of the cochlear frequency channels to separate the target stream from the background interference.

Figure 1 depicts the block diagram for the FPGA implementation of the temporal coherence model. The cochlea receives the auditory input, and transforms the sound signal into a frequency spectrum. The cochlear output is passed on to the rate filters that perform a temporal multi-rate analysis, integrating the history of cochlear channel responses. An attention signal, which is a part of the target stream influences stream formation by initiating binding. A correlation matrix that measures the similarity of auditory responses across channels needs to be computed, but this is computationally very costly for FPGA implementation. To ease the computational burden of this calculation, we use the attention signal to choose particular channels of interest. Pair-wise correlation of all channels is computed with the attention signal, which acts as a mask in stream reconstruction and is referred to as correlation column. Negative values of correlation coefficient indicate that other tones are highly uncorrelated with the attention signal. This allows us to compute only a single column of the correlation matrix, which represents correlation of the attention channel with all other channels. This single column is referred to as a mask. Finally, the mask is used to separate the target stream from the background interference and the filtering representations are converted back to the acoustic domain. Each of the modules of the architecture is described in detail below.

#### Cochlea

The cochlea functions as a front-end analyser for sound by transforming the sound input into a frequency spectrum. We utilize the CAR-FAC model of the cochlea (Lyon, 2011) in our system, as its speed and efficiency is superior to the more conventional parallel filter bank approach (Lyon, 1998). The asymmetric resonators in the cascade of asymmetric resonators (CAR) are quasi-linear transfer functions that model the motion of the BM. The outer hair cell module provides dynamic non-linearity or fast-acting compression (FAC). The inner hair cells are encoded using sigmoidal or half-wave rectification function, and introduce non-linearity in the outputs of the CAR. They function to connect the mechanical waves on the BM to neural signals on the auditory nerve.

**Figure 2:**
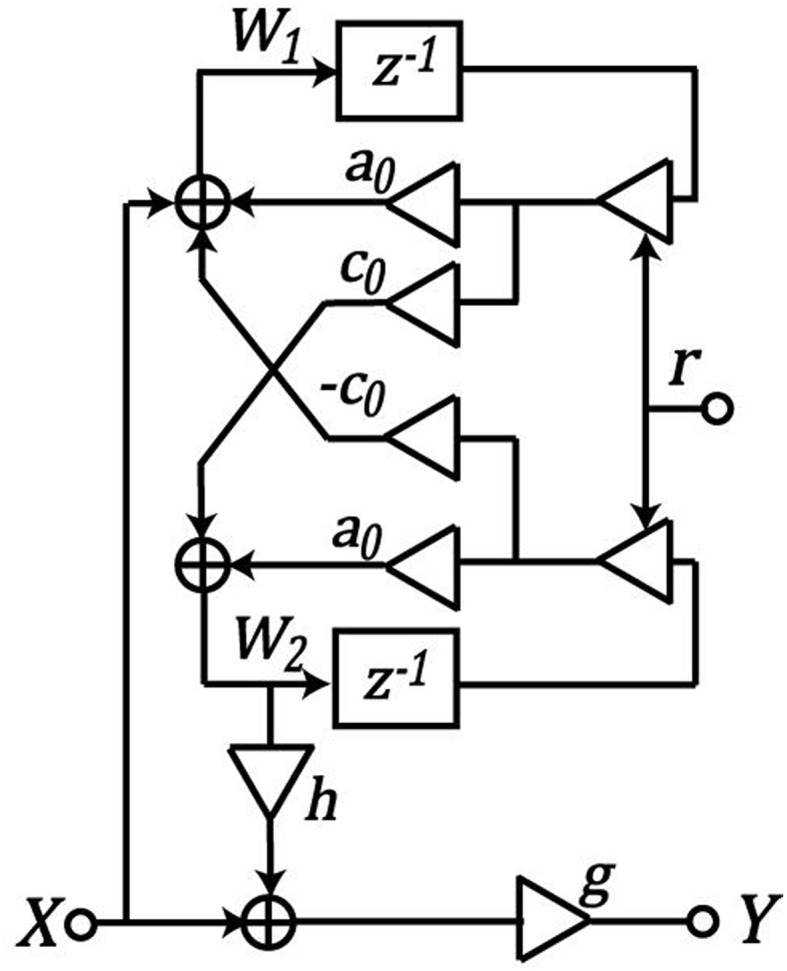
**Two-pole–two-zero filter in the CAR model**. *a*_**0**_ and *c*_**0**_ are functions of position *x* along the cochlea. *h* controls the difference between zeros and the pole frequency, *g* is used to adjust the overall gain. *X* represents the input signal, *Y* represents the output signal, and *W*_**1**_ and *W*_**2**_ are internal state variables. Figure adapted from Thakur et al. (2014a).

Biquadratic filters represent asymmetric resonators in the CAR model of the BM. The number of filter sections and their coefficients are optimized to match a linearised model of the cochlea. The filter poles are equally spaced along the length of the cochlea. For a normalized position *x* along the cochlea, the pole frequency, *f*, is obtained using the Greenwood function (Greenwood, 1990):

$$\begin{array}{l} f=165.4\left(10^{2.1x}-1\right) \end{array}$$

where, *x* varies from 0 at the apex of the BM, to 1 at its base. Figure 2 shows a biquadratic filter section. Parameters *a*_0_ and *c*_0_ are functions of position *x*, and represent the analog pole position in the zero-damping case. An explicit parameter, *r*, can be modulated to vary the pole and zero radius in the *z* plane, thus modulating the damping factor. The relationship between these parameters is given using the following equations:

$$\begin{array}{l} a_{0}=\cos\left(\theta_{R}\right)=a∕r\\ c_{0}=\sin\left(\theta_{R}\right)=c∕r \end{array}$$

where, θ_*R*_ is the normalized pole ringing frequency or pole angle in the *z* plane. The transfer function is given as:

$$\begin{array}{l} \frac{Y}{X}=g\frac{z^{2}\;+\left(-2a_{0}\;+\;hc_{0}\right)rz\;+\;r^{2}}{z^{2}-2a_{0}rz\;+\;r^{2}} \end{array}$$

The *h* coefficient controls the difference between zeros and the pole frequency, and the *g* coefficient is used to adjust the overall gain. The zeros will be at the same radius *r* as the poles, if *h* is small enough that the zeros remain complex. For high-frequency channels, cos θ_*R*_ < 0. In that case:

$$\begin{array}{l} h_{0}<\frac{2\;+\;2a_{0}}{c_{0}} \end{array}$$

To get unity gain at DC, we can solve for *g*:

$$\begin{array}{l} g=\frac{1-2a_{0}r\;+\;r^{2}}{1-\left(2a_{0}-hc_{0}\right)r\;+\;r^{2}} \end{array}$$

The combination of cascaded stages creates a family of filters at the output taps between the stages. The resulting filters may have high peak gains, depending on the stage damping parameters.

We have previously implemented the CAR module of the CAR-FAC model in FPGA, which represents the cochlear basilar membrane (BM) (Thakur et al., 2014a). Here we have added a simplified inner hair cell (IHC) model using a half-wave rectifier at the output of the CAR filters followed by two first order low-pass filters with a 8 kHz cut-off frequency, to generate an approximate neural activity pattern. Although this is a very simplified model of the IHC function, it suffices for our application.

#### Rate filter

While the cochlea is selective only for the frequency content of sound, cortical auditory neurons are additionally selective for temporal modulations found in natural sounds (Kowalski et al., 1996; Theunissen et al., 2000; Lu et al., 2001; Escabí et al., 2003; Woolley et al., 2005). Hence, a temporal analysis of the auditory spectrogram generated by the cochlea is carried out with multi-range dynamics covering a frequency range of 2–16 Hz. For this, the output of each cochlear channel is connected to a rate filter. In our FPGA design, we have implemented only a 4 Hz rate filter using two sets of low pass filters (LPF) and high pass filters (HPF), each with the same cut-off frequency of 4 Hz and connected in series to obtain steeper slopes. The LPF and HPF are represented using the following equations:

$$\begin{array}{l} bpl_{t}=(\left(1-c_{l}\right)\;*\;bpl_{t-1}\;+\;c_{l}\;*\;\vartheta_{t}\\ bph_{t}=(\left(1-c_{h}\right)\;*\;bph_{t-1}\;+\;c_{h}\;*\;\left(bpl_{t}\;-\;bph_{t-1}\right) \end{array}$$

where, ϑ_t_ is input to the rate filters coming from the cochlea at time *t. bpl* and *bph* represent the LPF and HPF function, respectively. *c*_*l*_ and *c*_*h*_ denote the coefficients for LPF and HPF corresponding to a cut-off frequency of 4 Hz, respectively, and are given by:

$$\begin{array}{l} c_{l}=c_{h}=2\pi \left(\frac{4}{f_{s}}\right) \end{array}$$

where, *f*_*s*_ is the sampling frequency.

#### Attention signal and mask for reconstruction

Attention is a cognitive process that allows one to focus on a group of features of an auditory stimulus. This enhances their relative amplitudes as compared to unattended stimuli, thus playing an important role in auditory stream perception and segregation (Snyder et al., 2006; Bidet-Caulet et al., 2007). It has been shown that there are attention-dependent changes in the spectro-temporal receptive fields of the auditory cortex, such as frequency selective enhancement (Fritz et al., 2003, 2007). Additionally, attention can influence streaming by modulating the temporal coherence of neural populations (Niebur et al., 2002). Attention is an exclusive feature present only in the target stream and it is used computationally to facilitate identification of the target stream.

The correlation vectors are computed as the product of the rate filter channels with the attention channels, and these vectors act as a mask. Only the instantaneous correlation across all pairs of channels is considered. Currently, we have implemented only the 4 Hz rate filter. In the case of multi-rate filters, we sum all the correlation coefficients of each cochlear channel and zero the negative coefficients of the resultant vector, which represents the mask. Usage of an attention signal eases the computational burden of this calculation, otherwise we would have to calculate the complete correlation matrix followed by decomposition into principle components using a non-linear auto-encoder (Krishnan et al., 2014). The computed mask is used for the reconstruction or segregation of the speech of interest. Since the time-scale of the rate filters is very slow (<20 Hz), and FPGA processing is very fast (~hundreds of MHz), we are able to use a single rate filter of 4 Hz across all the channels using a time-multiplexing technique.

#### Stream reconstruction

In stream reconstruction, the target stream present in the input auditory stimulus is resynthesised computationally using the output of early auditory and cortical stages. A detailed mathematical explanation of stream reconstruction is published by Chi et al. (2005). It should be noted that reconstruction of sound is not a biological process, but for sound segregation applications we need to reconstruct the target sound. In this final stage, point to point multiplication of the formed mask with the output of the BM channels of the cochlea is carried out to segregate the target stream from the background, and to reconstruct the stream. This process would require many multipliers, but our FPGA implementation requires only one multiplier, as we are using a time-multiplexing technique. Rate filtering introduces some latency and each BM filter also introduces a different phase delay. Currently, we are using a 100 MHz system clock. The BM block introduces a delay of 49 clock cycles (490 ns) and the total delay introduced by the IHC and the rate filter is 45 clock cycles (450 ns). These could be compensated for by delaying each BM output channel by an appropriate amount before reconstruction, but in the current implementation, we have not done this as the quality of the reconstructed signal without delay compensation is good enough for our purpose.

### FPGA implementation

**Figure 3:**
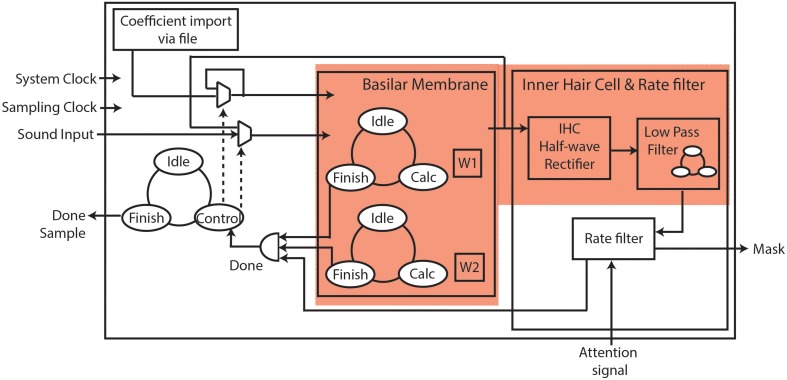
**System Architecture for FPGA implementation**. The system includes a basilar membrane block, and an inner hair cell and rate filter block. Each block is only instantiated once, but is time multiplexed to create 70 instances in our system. The cochlea is shown using a red background.

First, we simulated a software floating-point implementation of the model in Python. Next, we adapted the Python code for fixed-point implementation, and determined the word length of the input, output, and internal variables required for FPGA implementation without loss of accuracy. The system architecture for the hardware implementation is shown in Figure 3. Here, we have used time-multiplexing to share the hardware resources on the FPGA. We have implemented a single hardware block as shown in Figure 3, and reused it for all the 70 filter sections, given an audio sampling clock of 8 KHz and a system clock of 100 MHz (Thakur et al., 2014b). The cochlear filter section that is processed at a particular time is determined by a global state machine. The latter also controls the coefficients and data for the filter section. For each filter section, the coefficients *a, c, g*, and *h* are calculated externally, and uploaded into FPGA memory from a file at the start of execution. Each input sound sample passes via the global state machine to the BM for processing. Two parallel state machines are contained in the BM block. These control and calculate internal variables W1 and W2, which are further used to calculate the transfer function, *Y/X*. A delay element (*z*^−1^ block in Figure 2) requires the variables, W1 and W2, to be stored for each filter section. The output of the BM is passed on to the inner hair cell and rate filter block for each filter section, as explained in Section Rate Filter. The output of each cycle of operation of one filter section is passed to the global state machine. This serves as the input for the next filter section stage, resulting in a cascading of the filter sections. The completion of processing of an input sample by one filter, inner hair cell and rate filter section is denoted as “Done,” while “Done_Sample” by the global state machine denotes the completion of processing by all the filter sections including inner hair cell and rate filter. We have successfully implemented the proposed system on an Altera Cyclone V FPGA (on a Terasic Cyclone GX starter kit) with the utilization area as shown in Table 1.

**Table 1:** **Device utilization Altera Cyclone-V 5CGXFC7C7F23C8**.

**Adaptive Logic Modules (ALMs)**	**Total registers**	**DSPs**
1793/56480 (3%)	3899	10/156 (6%)

## Results

Here, we present test results for the performance of the system. We first tested the model on typical auditory stimuli (single and complex tones) widely used to study the perceptual formation of auditory streams. Further, we tested the model on complex speech sound for speaker separation. The results presented in Sections Segregation of Streams from Alternating-tone Sequence and Segregation of Streams from Alternating Complex Tones were obtained from hardware implementation, and those in Section Segregation of Speech from Mixtures were obtained from simulation of the model in software, which is a replica of our hardware model. The performance of the model is quantified by comparing the original separate streams to the segregated stream. The signal-to-noise ratio (SNR) is computed as:

(1) $$\begin{array}{r} \text{SNR\_segregated\_stream }=\;10log\left(\frac{{\left|S1*O1\right|}^{2}}{{\left|S1*O2\right|}^{2}}\right) \end{array}$$

(2) $$\begin{array}{r} \text{SNR\_mixture }=\;10log\left(\frac{{\left|M*O1\right|}^{2}}{{\left|M*O2\right|}^{2}}\right) \end{array}$$

where, *S1* is the segregated (output) stream; *O1* and *O2* are the original separate streams; and *M* is the mixture stream provided as the input. The “*” operator represents the dot product of the two sound vectors.

### Segregation of streams from alternating-tone sequence

**Figure 4:**
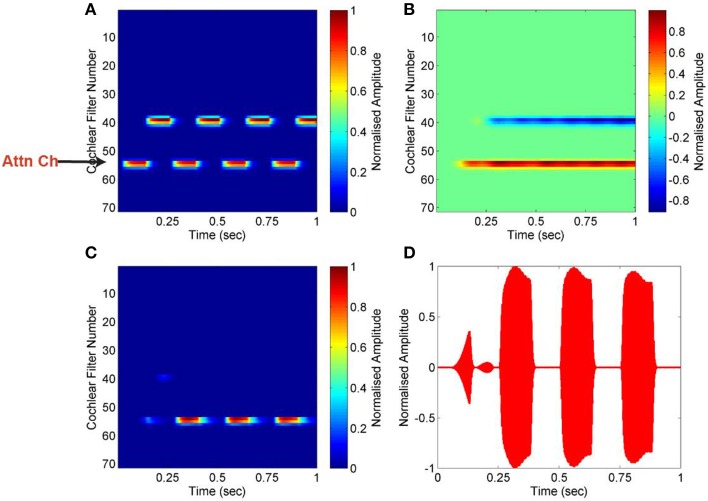
**Segregation of alternating-tone sequence. (A)** Auditory spectrogram of the input mixture of two alternating pure tones (440 and 1000 Hz). A low cochlear filter number represents high frequency, and *vice versa*. One particular channel, of frequency 440 Hz, is used as the attention signal (Attn Ch). (**B)** Pair-wise correlation of all channels are computed using the attention signal. The negative value of correlation coefficient (*blue*) indicates that the second tone is highly uncorrelated with the attention signal. (**C,D)** The targeted stream is segregated, and reconstructed using mask which is created based on the attention signal, shown as a spectrogram in **(C)** and plot in (**D)**.

An alternating-tone sequence is composed of two continuously repeated pure tones of different frequencies, A and B. Such a sequence is commonly used in studies of auditory stream segregation. The frequency separation between the two tones, Δ*f*, and the inter-tone interval, Δ*T*, determine the percept evoked by such sequences. If Δ*f* is small and Δ*T* is long, the sequence is perceived as a single stream of tones alternating in frequency (ABAB). This phenomenon is known as temporal coherence (Van Noorden and Schouten, 1975). On the contrary, if Δ*f* is large and Δ*T* is short, the sequence is perceived as two separate streams of tones of constant frequencies (A's and B's). This phenomenon is known as stream segregation. We have used a sequence composed of frequencies 440 and 1000 Hz, with a presentation rate (Δ*T*) of 4 Hz. Figure 4 shows the result for this alternating-tone sequence. The input mixture is transformed into an auditory spectrogram using cochlea as described in Section Cochlea, and a particular channel that is a feature of the targeted stream is used as attention signal. Pair-wise correlation of all channels is computed with the attention signal which we refer as correlation column. The negative value of correlation coefficient indicates that the tone of frequency 1000 Hz is highly uncorrelated with the attention signal. The targeted stream of 440 Hz tone is segregated, and reconstructed using mask which is created based on the attention signal. The effectiveness of segregation is measured using Equations (1) and (2). The SNR for the segregated stream is calculated as 91 dB compared to the input mixture of two simple tones, mixed at 0 dB.

### Segregation of streams from alternating complex tones

**Figure 5:**
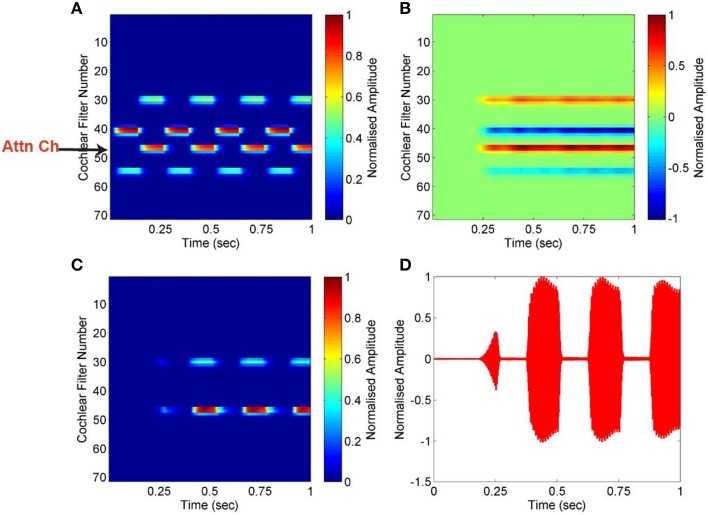
**Segregation of alternating complex tones. (A)** Auditory spectrogram for an input mixture of complex tones [(300, 900 Hz) and (600, 1500 Hz)]. Low cochlear filter number represents high frequency, and *vice versa*. The channel corresponding to frequency of 600 Hz is used as attention signal (Attn Ch) and is marked with an arrow. (**B)** Pair-wise correlation of all channels is computed using the attention signal. Negative values of correlation coefficient (*blue*) indicate that other tones are highly uncorrelated with the attention signal. (**C,D)** The targeted stream (600, 1500 Hz) is segregated and reconstructed using mask generated based on the attention signal, shown as a spectrogram in (**C)** and plot in (**D)**.

Here, we have used a sequence of two complex tones alternating with a presentation rate of 4 Hz. This presentation rate lies within the range (2–20 Hz) of the presentation rate of auditory signals over which auditory stream formation takes place in the brain (Fishman et al., 2004; Chakalov et al., 2013). The first complex tone is a mixture of pure tones of frequencies 300 and 900 Hz, and the second complex tone consists of tones of frequencies 600 and 1500 Hz. The results are shown in Figure 5. The tone with frequency of 600 Hz is used as the attention signal. As this tone is temporally coherent with the tone of frequency 1500 Hz, all two tones become segregated as one stream. It can be seen in Figure 5 that all frequencies comprising the first mixture—600 and 1500 Hz, show positive correlation coefficient because they are temporally coherent with the attention signal. In contrast, the other complex tone of frequencies 300 and 900 Hz show negative correlation coefficients, suggesting that they belong to a different acoustic source and are incoherent with the attention signal. The targeted stream is segregated and reconstructed using the mask generated based on the attention signal. The effectiveness of segregation is measured using Equations (1) and (2). The SNR for the segregated stream is calculated as 77 dB compared to the input mixture of two complex tones, mixed at 0 dB.

### Segregation of speech from mixtures

**Figure 6:**
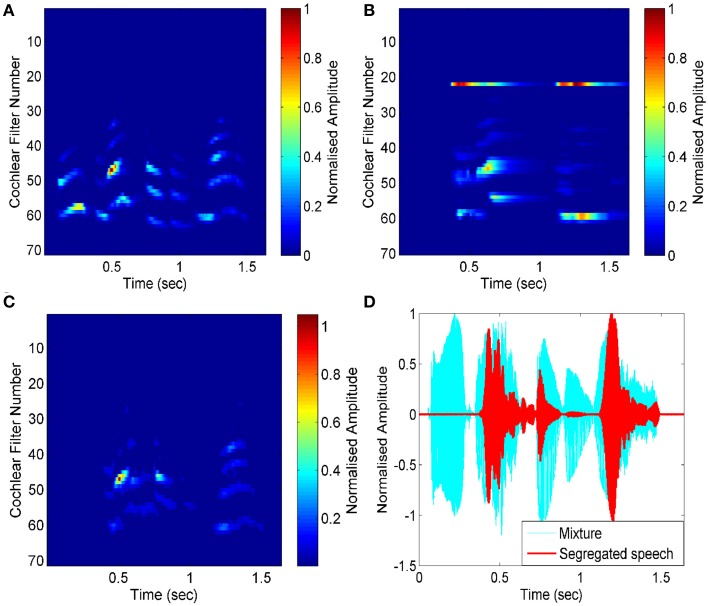
**Software simulation for segregation of speech from mixture. (A)** Auditory spectrogram for an input mixture of two female utterances (“good morning” and “game over”) is transformed into an auditory spectrogram. The envelope of the target speech (“good morning”) acts as the attention signal. **(B)** Pair-wise correlation of all channels are computed with the attention signal (channel number 23). **(C)** The segregated target streams are shown in the spectrogram. **(D)** Input mixture (cyan graph) and segregated speech (red graph) are shown.

Here, we have used a mixture of two female utterances, one saying “good morning” and the other saying “game over.” The target speech is the female speech corresponding to “good morning.” Our system needs an attention signal, which should be present exclusively in the target speech. Normally, this might be channels tuned roughly near the pitch range, or location responses sensitive to the approximate direction of the target speaker. By using these channels, we can simply use their coherently-modulated power as the cue to the presence of the target speaker. Here, to simulate these extra computations, we have used the envelope (power) of the target speech to act as the attentional signal. The results are shown in Figure 6. Pair-wise correlation of all channels is computed with this simulated attention signal, resulting in the necessary mask in stream reconstruction. As shown in Figure 6, we are able to segregate the target speech signal from the speech mixture efficiently. The effectiveness of segregation is measured using Equations (1) and (2). The SNR for the segregated stream is calculated as 55 dB compared to the input mixture of the two female utterances, mixed at 0 dB.

## Discussion

In this work, we have adapted a temporal coherence-based computational model of auditory scene analysis for neuromorphic hardware implementation in FPGA. We have validated our system by testing various sound stimuli such as alternating-tone sequence, alternating-harmonic complexes and mixtures of speech. We show that our FPGA architecture can successfully segregate sound streams in all these cases (see Supplementary Material). Our system implements a neuromorphic model of the segregation of sound sources in real-time. The system is easily scalable to incorporate higher number of cochlear channels and rate filters, and thus may serve as a feasible solution to the cocktail party problem.

The temporal coherence algorithm differs from other computational systems of auditory stream segregation in its close correspondence to the neurobiological cortical mechanisms of hearing (Shamma et al., 2011). The algorithm also requires no prior information or training on the sources, and can gracefully incorporate and benefit from attention as a criterion for stream segregation. Research has shown that attention plays an important role in segregation by enhancing the perception of a particular stream over others in the auditory scene (Hillyard et al., 1973; Tiitinen et al., 1993; Bidet-Caulet et al., 2007; Elhilali et al., 2009b). Here, we utilize an attention signal as a means to separate target sound from background noise.

In our previous work, we have implemented a cochlear model and demonstrated its ability to process sound in real-time (Thakur et al., 2014b). We have now integrated this cochlear implementation with the temporal coherence model, and this system is a novel prototype for multi-talker speech separation and recognition. Future work will aim to extend the existing model for the segregation of complex speech signals to incorporate various cues such as pitch, frequency, timbre, and spatial location etc. Additionally, the limitations of the current system will be addressed in the future. For example, attention signal is only one of the means to identify a feature of interest in the target stream for segregation. We will incorporate additional features that will make the system more robust to segregate sound. The correlation vector in our system is implemented using a multiplier. This is a simplified model for the auditory cortex, which will be improved by using spike-based computation to group coherent features. Finally, we could also expand the model to explore the effects of switching attention channel between two streams and look at how this switching affects the representation of streams.

Overall, our FPGA implementation of the temporal coherence algorithm establishes that it is feasible to develop a hardware system that can segregate sound sources in real-time. Our FPGA implementation is area efficient, since it reuses a single hardware block for all the filter sections. Our system will have several applications, such as robust front-end processors for automatic speech recognition.

### Conflict of interest statement

The authors declare that the research was conducted in the absence of any commercial or financial relationships that could be construed as a potential conflict of interest.
